# Development and internal validation of a predictive model for intrapartum hypertension: a retrospective case-control study

**DOI:** 10.1186/s12884-026-08952-2

**Published:** 2026-03-17

**Authors:** Yihan Lu, Ruofan Kong, Ruoyun Wei, Lizhou Sun, Xiaotong Tang, Yuanyuan Zhang

**Affiliations:** 1 Department of Obstetrics and Gynecology, the First Affiliated Hospital of Nanjing Medical University, Nanjing, 210029 Jiangsu China; 2 Department of Obstetrics and Gynecology, Women’s Hospital of Nanjing Medical University, Nanjing Women and Children’s Healthcare Hospital, Nanjing, 210004 Jiangsu China

**Keywords:** Intrapartum hypertension, Maternal and neonatal outcomes, Ppredictive model, Case-control study, Risk factors

## Abstract

**Background:**

Intrapartum hypertension is associated with adverse outcomes, yet tools for its prediction are limited. This study aimed to develop a predictive model using routine clinical data.

**Methods:**

This retrospective case-control study included 200 women, comprising 100 with intrapartum hypertension and 100 matched controls. We developed a predictive model by first screening candidate variables through univariate analysis. Variables that showed significant differences were then entered into a multivariate logistic regression model to identify independent predictors. The model’s performance was evaluated using the area under the receiver operating characteristic curve (AUC). Internal validation was conducted via the bootstrap method with 500 resamples to correct for optimism and to estimate a more generalizable AUC. Intrapartum hypertension was defined as a systolic blood pressure ≥ 140 mmHg or a diastolic blood pressure ≥ 90 mmHg during labor, confirmed on two separate occasions. Risk factors were identified using univariate and multivariate logistic regression, and predictive performance was assessed by ROC analysis.

**Results:**

The case group exhibited higher rates of cesarean delivery (14% vs. 4%), higher peak blood pressure, and greater neonatal birth weight (all *P* < 0.05). Independent risk factors identified were elevated lactate dehydrogenase (LDH), higher admission weight, gestational diabetes mellitus, proteinuria in the second trimester or antenatal period (all *P* < 0.05). The final multivariate model achieved an AUC of 0.832. After bootstrap internal validation, the optimism-corrected AUC was 0.815.

**Conclusion:**

A model incorporating five routinely available clinical parameters (including lactate dehydrogenase level, admission weight, gestational diabetes mellitus, and second-trimester or antenatal proteinuria) effectively identifies parturients at high risk for developing intrapartum hypertension. This tool can facilitate early, targeted monitoring in clinical practice.

**Supplementary Information:**

The online version contains supplementary material available at 10.1186/s12884-026-08952-2.

## Introduction

Hypertensive disorders of pregnancy (HDP) are defined by new-onset hypertension (systolic blood pressure ≥ 140 mm Hg and/or diastolic blood pressure ≥ 90 mm Hg) after 20 weeks of gestation. This condition may be accompanied by proteinuria or other signs of end-organ dysfunction, such as thrombocytopenia or abnormal liver or kidney function [1, 2]. Although the exact pathogenesis of preeclampsia is not fully understood, current hypotheses emphasize impaired spiral artery remodeling, oxidative stress, and genetic factors [3–5]. To date, delivery remains the only definitive treatment.

Intrapartum hypertension represents a severe clinical manifestation of HDP and significantly affects both maternal and neonatal outcomes [6, 7]. Its development is closely linked to the physiological stress of labor, which includes frequent uterine contractions, maternal anxiety, and marked hemodynamic changes. With approximately 300–500 mL of blood entering the systemic circulation per contraction, these factors collectively promote further elevation in blood pressure [8, 9]. When superimposed on pre-existing hypertensive disorders, such stresses may lead to cardiovascular decompensation and serious complications, including eclampsia and maternal stroke. A rapid rise in blood pressure during labor can also rupture placental basal membrane vessels, resulting in placental abruption. This may progress to severe intrapartum hemorrhage and disseminated intravascular coagulation (DIC) [10, 11]. The strong association between acute hypertensive episodes and placental abruption is well established, with studies identifying hypertensive disorders as a leading risk factor for this catastrophic obstetric complication [12]. A large cohort study published in the *European Journal of Obstetrics & Gynecology and Reproductive Biology* in 2011 demonstrated that HDP more than doubled the risk of placental abruption, and acute intrapartum hypertension further increased this risk [13].

However, the management of acute onset or exacerbation of hypertension during labor is uniquely challenging due to the concurrent physiological stress and the limited therapeutic windows available. Despite substantial knowledge regarding risk factors for general HDP, models specifically designed and validated to predict this acute intrapartum deterioration using routinely available clinical data are still lacking.

Currently, predictive indicators for intrapartum hypertension remain inadequately characterized worldwide, with most evidence derived from studies on broader hypertensive disorders. Beyond traditional risk factors, pregnancies conceived via assisted reproductive technology (ART) have been associated with an altered risk profile for adverse outcomes, including hypertensive disorders [14]. A brief review of existing data specific to intrapartum hypertension [6, 7] confirms its clinical importance but also highlights the absence of validated, multivariate prediction tools that comprehensively integrate such diverse risk factors.

Intrapartum hypertension, as a temporal variant of gestational hypertension/preeclampsia in the ACOG (2020) [15] and ISSHP (2018) [16] HDP classification systems, is defined as new-onset hypertension (SBP ≥ 140 mmHg and/or DBP ≥ 90 mmHg) during labor in a previously normotensive pregnant woman; when accompanied by proteinuria or end-organ dysfunction, it is classified as intrapartum-onset preeclampsia. This study focuses on intrapartum hypertension as a timing-specific manifestation of conventional HDP, rather than a distinct nosological entity.

The primary objective of this study was to identify independent predictors and develop a multivariate model for intrapartum hypertension in a cohort of pregnant women who were normotensive before labor onset. Accordingly, this study aimed to enroll 100 women with intrapartum hypertension and an equal number of normotensive women as controls. We collected and analyzed clinical data to assess the impact of intrapartum hypertension on maternal and neonatal outcomes, identify potential predictive factors, and establish a basis for early clinical identification and intervention.

## Method

### Study design and participants

This study was conducted in accordance with the principles of the Declaration of Helsinki. The study protocol was approved by the Institutional Review Board (IRB) of The First Affiliated Hospital of Nanjing Medical University (Approval Number: 2021-SR-055). This approval is an umbrella protocol that explicitly covers retrospective data collection for pregnancy outcome studies conducted in the hospital from January 2021 to December 2025, including the July 1, 2023, to December 31, 2024, study period of the present research. Due to the retrospective nature of the study, the same IRB waived the requirement for informed consent.

**Fig. 1:**
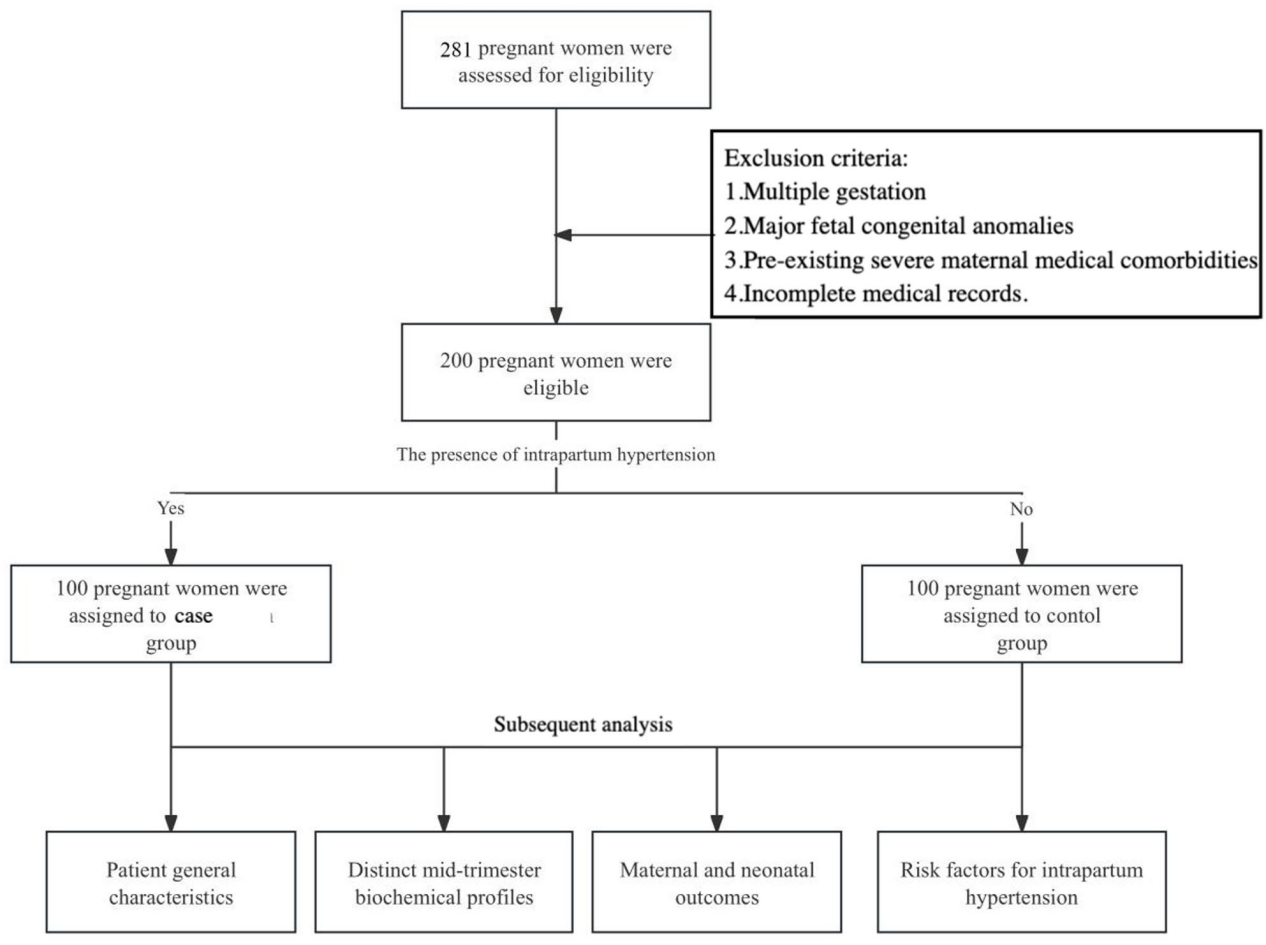
Flowchart of patient selection and grouping

As shown in Fig. 1, we assessed for eligibility all pregnant women who delivered at the hospital between July 1, 2023, and December 31, 2024. Inclusion criteria were: (1) singleton gestation; (2) complete prenatal examination and delivery outcome records; and (3) normotensive status before labor onset. Normotension was defined as the absence of any documented diagnosis of chronic hypertension, gestational hypertension, preeclampsia, or eclampsia, and no antenatal blood pressure reading ≥ 140/90 mm Hg from 20 weeks of gestation until the onset of labor. This criterion aimed to select women who developed new-onset hypertension specifically during labor and delivery. Exclusion criteria were: (1) multiple gestation; (2) major fetal congenital anomalies; (3) pre-existing severe maternal comorbidities (e.g., chronic kidney disease, autoimmune diseases); (4) a documented diagnosis of chronic hypertension (regardless of antihypertensive medication use or blood pressure control during pregnancy), gestational hypertension, preeclampsia, or eclampsia at any time during the current pregnancy; and (5) incomplete medical records.

A total of 281 women were initially assessed during the study period. Of these, 181 were identified as having experienced intrapartum hypertension. After applying the inclusion and exclusion criteria, 100 women (55.2%) were consecutively enrolled as the case. The remaining 81 women were excluded due to incomplete medical or laboratory data. Baseline demographic comparisons between included and excluded intrapartum hypertension cases (age, gestational age at delivery, mode of delivery) are presented in Supplementary Table S1, showing no statistically significant differences (all *P* > 0.05). The control group comprised 100 normotensive women selected from the remaining eligible population. To control for confounding, multivariable logistic regression was performed with adjustment for maternal age, gestational age at delivery, admission weight, parity, and gestational diabetes mellitus (GDM) as covariates; propensity score matching was not used for group matching. Final group allocation was based on the presence or absence of hypertension diagnosed during labor and delivery.

### Definition and measurement of intrapartum hypertension

In this study, intrapartum hypertension was defined as new-onset systolic blood pressure (SBP) ≥ 140 mm Hg and/or diastolic blood pressure (DBP) ≥ 90 mm Hg, recorded on at least two consecutive occasions at least 15 min apart, during the period from the onset of regular uterine contractions (established labor) until two hours after placental delivery. Severe-range hypertension (SBP ≥ 160 mm Hg and/or DBP ≥ 110 mm Hg) was also documented as a subgroup for descriptive analysis but was included in the same intrapartum hypertension diagnostic criteria for univariate and multivariate statistical analyses, without separate stratification in the predictive model development.

Blood pressure measurement followed a standardized protocol to minimize bias. All readings were obtained using calibrated automated oscillometric devices (Omron HEM-7320, an upper-arm monitor manufactured by Omron Healthcare Co., Ltd., Kyoto, Japan) with appropriately sized cuffs. Cuff size was selected based on maternal upper arm circumference (22–26 cm for standard cuff, 27–34 cm for large cuff) in accordance with the manufacturer’s instructions. Measurements were taken with the parturient in a semi-recumbent or left lateral position during uterine quiescence (between contractions). The right arm was used consistently and supported at heart level. Blood pressure measurements were obtained after the parturient had rested for at least 5 min in a quiet environment to eliminate the influence of acute activity or anxiety. Hospital policy required blood pressure monitoring every 30–60 min during active labor, with more frequent measurements if hypertension was suspected. The use of labor analgesia (epidural) and oxytocin for induction or augmentation was recorded for both groups and was comparable between them (*P* > 0.05), ensuring that observed blood pressure differences were not attributable to these interventions. This definition and measurement approach aligns with diagnostic guidelines from the International Federation of Gynecology and Obstetrics and the American College of Obstetricians and Gynecologists.

### Verification of normotensive status before labor

To rigorously adhere to the inclusion criterion of “normotensive status before labor onset,” we performed a comprehensive review of all prenatal medical records for each participant from 20 weeks of gestation until hospital admission for delivery. Any recorded systolic blood pressure ≥ 140 mm Hg or diastolic blood pressure ≥ 90 mm Hg during this period resulted in exclusion. This process was designed to minimize the inclusion of women with undiagnosed chronic or gestational hypertension that manifested prior to the onset of labor.

### Data collection

Clinical data for the 200 pregnant women were systematically retrieved from the hospital’s electronic medical record system for retrospective analysis. The collected information included the following categories:

1. General patient information: Maternal age, parity, height, pre-pregnancy weight, admission weight at delivery, pre-pregnancy body mass index (BMI), admission BMI, gestational weight gain, mode of conception (spontaneous or assisted reproductive technology), family history of hypertension, and comorbidities including gestational diabetes mellitus (GDM) and hypothyroidism.
2. Second-trimester laboratory parameters: Urinary protein levels (qualitative and semi-quantitative analysis; positive defined as trace or higher) were measured at two time points: gestational urinary protein (24–28 weeks of gestation) and antenatal urinary protein (32–36 weeks of gestation); the kappa agreement coefficient between the two variables was 0.52 (moderate agreement), and the variance inflation factor (VIF) was 1.046 (no significant collinearity). Serum levels of lactate dehydrogenase (LDH), albumin (ALB), total cholesterol (TC), high-density lipoprotein (HDL), and low-density lipoprotein (LDL). All laboratory analyses were performed according to the hospital’s standard operating procedures.
3. Delivery and outcome measures: Mode of delivery (vaginal or cesarean section), peak systolic and diastolic blood pressures recorded during labor, gestational age at delivery, occurrence of postpartum hemorrhage, neonatal birth weight, preterm birth (defined as delivery before 37 weeks of gestation), 1- and 5-minute Apgar scores (with the clinically standard threshold of < 7 for identifying neonates requiring medical attention), and neonatal intensive care unit (NICU) admission.

Specific laboratory parameters (e.g., LDH, ALB, TC, HDL, and LDL) were selected based on their established pathophysiological associations with endothelial dysfunction, oxidative stress, and lipid metabolism disorders, which are implicated in the development of HDP.

### Statistical analysis

Data were analyzed using SPSS Statistics version 26.0 (IBM Corp., Armonk, NY, USA). All continuous variables were first tested for normality using the Shapiro-Wilk test and Q-Q plot analysis; no continuous variables were subjected to variable transformation or piecewise processing, as the Mann–Whitney U test (for non-normal distribution) and independent-samples t test (for normal distribution) were selected for comparative analysis based on the original distribution characteristics, which avoided information loss caused by artificial variable processing. Continuous variables with normal distribution are presented as mean ± standard deviation and were compared using the independent-samples *t* test. Continuous variables with non-normal distribution are expressed as median (interquartile range) and were compared using the Mann–Whitney *U* test. Categorical data are presented as number (percentage) and were compared using the chi-square test or Fisher’s exact test, as appropriate. Given the exploratory nature of the univariate analyses and the subsequent multivariate modeling, no adjustment for multiple comparisons was applied; however, this limitation should be considered when interpreting the findings.

Variables that showed statistically significant differences in univariate analyses were entered into a multivariate logistic regression model to identify independent risk factors for intrapartum hypertension. Variable selection was performed using Lasso regression, which excluded primiparity from the final model due to L1 regularization penalty. A variance inflation factor (VIF) screening was performed to mitigate multicollinearity among highly correlated anthropometric variables (e.g., admission weight, pre-pregnancy weight, various BMI measures, and weight-to-height ratio). Only admission weight was retained in the final model as the representative anthropometric variable, as it demonstrated the strongest univariate association and its VIF was below 2.5, indicating no substantial multicollinearity with other retained predictors. Results are reported as odds ratios (OR) with 95% confidence intervals (CI).

The final multivariate logistic regression model underwent internal validation with 500 bootstrap resamples to correct for overoptimism. The optimism-adjusted area under the curve (AUC) and the calibration slope were calculated accordingly.

Missing data for any candidate predictor variable were addressed through complete-case analysis; individuals with missing key variables were excluded from the logistic regression modeling.

No formal a priori sample size calculation was performed due to the retrospective nature of the study and the lack of preliminary data on intrapartum hypertension prediction at the time of study design.

## Results

### Participants

All pregnant women who delivered in the Department of Obstetrics at The First Affiliated Hospital of Nanjing Medical University between July 1, 2023, and December 31, 2024, were screened for eligibility. During the study period, 181 women were diagnosed with intrapartum hypertension. From this group, 100 women (55.2%) met the inclusion criteria and were consecutively enrolled as the case group. The remaining 81 (44.8%) were excluded, primarily due to incomplete records (*n* = 45) or pre-existing hypertensive disorders (*n* = 36). Based on the presence or absence of intrapartum hypertension, participants were categorized into a case group (*n* = 100) and a control group (*n* = 100) consisting of normotensive women who met the same eligibility criteria.

The median maternal age was 30 years [interquartile range (IQR): 28.00–32.75] in the case group and 31 years (IQR: 28.00–33.00) in the control group. Comparative analysis of baseline characteristics showed that admission weight, admission BMI, gestational weight gain, and weight-to-height ratio were significantly higher in the case group than in the control group (all *P* < 0.05). Pre-pregnancy weight and BMI were also higher but were not carried forward into the multivariate model due to collinearity concerns (see Statistical Analysis). Additionally, the case group had a significantly higher proportion of primiparous women (87% vs. 63%, *P* < 0.001) and a higher incidence of GDM (29% vs. 13%, *P* = 0.005). These residual differences were adjusted for in the multivariate analysis. Detailed characteristics are presented in Table 1.

**Table 1:** General characteristics of the case group and the control group

Characteristics	Case Group (*N* = 100)	Control Group (*N* = 100)	U (χ༒)	*P*
Age, years [M (IQR)]	30.00 (28.00, 32.75)	31.00 (28.00, 33.00)	4717.00	0.487
Admission weight [M (IQR)]	75.50 (68.50, 83.30)	68.90 (62.74, 74.00)	2932.00	< 0.001^*^
Pre-pregnancy weight [M (IQR)]	60.00 (55.00, 68.95)	54.30 (49.40, 60.00)	3143.50	< 0.001^*^
Admission BMI [M (IQR)]	28.17 (26.45, 30.61)	25.64 (23.97, 28.13)	2855.00	< 0.001^*^
Pre-pregnancy BMI [M (IQR)]	22.86 (20.88, 25.34)	20.51 (19.14, 22.58)	3114.00	< 0.001^*^
Weight increment during gestational period [M (IQR)]	14.75 (11.53, 18.00)	12.85 (11.00, 15.90)	4022.50	0.017^*^
Weight increment / Pre-pregnancy weight [M (IQR)]	0.25 (0.18, 0.30)	0.24 (0.19, 0.29)	4966.00	0.934
Weight increment / Height [M (IQR)]	0.09 (0.07, 0.11)	0.08 (0.07, 0.10)	4105.50	0.028^*^
Parity, *n*(%)			15.360	< 0.001^*^
Primiparity	87 (87.0%)	63 (63.0%)		
Multiparity	13 (13.0%)	37 (37.0%)		
Assisted Reproductive Technology, *n*(%)			Fisher	1.000
YES	6 (6.0%)	6 (6.0%)		
NO	94 (94.0%)	94 (94.0%)		
Family History, *n*(%)			2.001	0.157
YES	54 (54.0%)	44 (44.0%)		
NO	46 (46.0%)	56 (56.0%)		
Comorbidity, *n*(%)				
GDM	29 (29.0%)	13 (13.0%)	7.715	0.005^*^
Hypothyroidism	21 (21.0%)	22 (22.0%)	0.030	0.863
Heart disease	2 (2.0%)	3 (3.0%)	Fisher	1.000

Continuous variables are presented as median (interquartile range) and were compared using the Mann–Whitney U test. Categorical variables are presented as number (percentage) and were compared using the chi-square test or Fisher’s exact test, as appropriate. A *P* value < 0.05 was considered statistically significant; significant results are marked with an asterisk (*)

### Distinct mid-trimester biochemical profiles

Analysis of second-trimester laboratory parameters revealed significant differences between the groups. Gestational urinary protein (24–28 weeks) : The proportion of negative gestational urinary protein results was significantly lower in the case group than in the control group (62.0% vs. 85.0%, *P* < 0.001), while the proportion of positive results was significantly higher (38.0% vs. 15.0%, *P* < 0.001). Antenatal urinary protein (32–36 weeks) : the case group showing a lower proportion of negative results (34.0% vs. 48.0%, *P* = 0.044) and a higher proportion of positive results (66.0% vs. 52.0%, *P* = 0.044).

The case group exhibited a significantly higher median LDH level compared to the control group (157.00 U/L vs. 142.00 U/L, *P* < 0.001) and a significantly lower median HDL level (1.92 mmol/L vs. 2.07 mmol/L, *P* = 0.025). No significant between-group differences were observed in albumin (ALB), TC, or LDL levels (all *P* > 0.05). Complete laboratory data are provided in Table 2.

**Table 2:** Laboratory tests in the second trimester of pregnancy in the case group and the control group

Characteristics	Case Group (*N* = 100)	Control Group (*N* = 100)	U (χ^༒^/t)	*P*
Gestational urinary protein, *n*(%)			13.580	< 0.001^*^
Negative	62 (62.0%)	85 (85.0%)		
Positive	38 (38.0%)	15 (15.0%)		
Antenatal urinary protein, *n*(%)			4.051	0.044^*^
Negative	34 (34.0%)	48 (48.0%)		
Positive	66 (66.0%)	52 (52.0%)		
LDH [M (IQR)]	157.00 (140.50, 170.00)	142.00 (132.25, 152.00)	2694.00	< 0.001^*^
ALB [M (IQR)]	37.05 (35.20, 38.80)	36.45 (34.93, 37.70)	4284.00	0.080
TC [M (IQR)]	5.83 (5.24, 6.65)	6.07 (5.18, 6.82)	4690.50	0.450
HDL [M (IQR)]	1.92 (1.64, 2.30)	2.07 (1.82, 2.36)	4080.50	0.025^*^
LDL (Mean ± SD)	3.42 ± 0.07	3.34 ± 0.79	-0.684	0.495

Continuous variables were presented as [M (IQR)] and compared using the *Mann-Whitney U* test, except for LDL which was presented as mean±standard deviation and compared using the independent-samples *t-test*. Categorical variables were presented as number (percentage) [*n* (%)] and compared using the *Chi-square test*. *P* < 0.05 was considered statistically significant, and significant results are marked with an asterisk (*). *Abbreviations*: *ALB* albumin, *HDL* high-density lipoprotein, *LDH* lactate dehydrogenase, *LDL* low-density lipoprotein, *TC* total cholesterol

### Maternal and neonatal outcomes

Comparative analysis revealed significant differences in several delivery outcomes. The cesarean delivery rate was significantly higher in the case group (14% vs. 4%; relative risk = 3.50, 95% CI: 1.20–10.22; *P* = 0.013). The relatively low overall cesarean rates (14% in cases, 4% in controls) likely reflect the low-risk profile of our study population, which excluded women with pre-existing hypertensive disorders and other major comorbidities. Additionally, institutional practices at our center emphasize trial of labor for eligible women, contributing to a comparatively high vaginal delivery rate. Peak systolic and diastolic blood pressures during labor were significantly elevated in the case group [systolic: 146.00 (144.00–151.75) vs. 125.00 (122.00–128.00) mm Hg; diastolic: 93.00 (89.00–97.00) vs. 78.00 (74.25–79.00) mm Hg; both *P* < 0.001]; these values are reported for descriptive characterization of group separation and are inherent to the diagnostic criteria for intrapartum hypertension, not representing independent outcome comparisons. The case group delivered at a slightly more advanced gestational age [39.71 (38.86–40.40) vs. 39.14 (38.29–39.82) weeks, *P* = 0.001], although all deliveries occurred at term. This small but statistically significant difference in gestational age (approximately 0.57 weeks, or 4 days) may have contributed to the observed difference in birth weight. Neonates in the case group had significantly higher birth weights [3400.00 (3162.50–3600.00) vs. 3225.00 (2962.50–3537.50) g, *P* = 0.011], with all values remaining within the normal range. No significant between-group differences were observed in the rates of postpartum hemorrhage, preterm birth, low Apgar scores (< 10 at 1 or 5 min), or NICU admission (all *P* > 0.05). Detailed outcomes are presented in Table 3.

**Table 3:** Outcomes of mothers and infants between the case group and the control group

	Case Group (*N*=100)	Control Group (*N*=100)	U(*X*^２^)	*P*
Parturition mode, *n*(%)			6.105	0.013^*^
Vaginal parturition	86 (86%)	96 (96%)		
Cesarean section	14 (14%)	4 (4%)		
Peak blood pressure during intrapartum period [M (IQR)]				
Systolic blood pressure	146.00 (144.00, 151.75)	125.00 (122.00, 128.00)	30.50	<0.001^*^
Diastolic blood pressure	93.00 (89.00, 97.00)	78.00 (74.25, 79.00)	217.50	<0.001^*^
Gestational age [M (IQR)]	39.71 (38.86, 40.40)	39.14 (38.29, 39.82)	3653.50	0.001^*^
Postpartum haemorrhage, *n*(%)			0.072	0.788
YES	8 (8%)	7 (7%)		
NO	92 (92%)	93 (93%)		
Neonatal outcome [M (IQR)]				
Neonatal weight	3400.00 (3162.50, 3600.00)	3225.00 (2962.50, 3537.50)	3959.50	0.011^*^
Preterm birth	2 (2%)	4 (4%)	Fisher	0.683
Apgar score (<7), *n*(%)				
At 1 min	5 (5%)	3 (3%)	0.649	0.521
At 5 min	1 (1%)	1 (1%)	Fisher	1.000
NICU, n(%)			2.079	0.149
YES	23 (23%)	15 (15%)		
NO	77 (77%)	85 (85%)		

Continuous variables were presented as [M (IQR)] and compared using the *Mann-Whitney U test*. Categorical variables were presented as number (percentage) [*n* (%)] and compared using the *Chi-square test* or *Fisher’s exact test*, as appropriate. *P* < 0.05 was considered statistically significant, and significant results are marked with an asterisk (*). *Abbreviations*: *BP* blood pressure, *NICU*, neonatal intensive care unit

### Risk factors for intrapartum hypertension

Variables that showed statistically significant differences in the univariate analyses (Tables 1 and 2) were entered into a multivariate logistic regression model with Lasso regression for variable selection to identify independent risk factors for intrapartum hypertension. Key baseline variables with residual imbalances (admission weight and BMI metrics) were also included as adjustment covariates. The VIF for each variable in the final model was examined to assess multicollinearity; all VIF values were below 5, indicating no significant multicollinearity. Primiparity was excluded from the final model by Lasso regression. The analysis identified elevated LDH (per 10 U/L increment: OR = 1.600, 95% CI: 1.290–1.985, *P* < 0.001) and increased admission weight (per 5 kg increment: OR = 1.537, 95% CI: 1.251–1.888, *P* < 0.001) as independent risk factors. Comorbid GDM was associated with a substantially elevated risk (OR = 4.265, 95% CI: 1.691–10.755, *P* = 0.002). Additionally, both second-trimester proteinuria (OR = 3.532, 95% CI: 1.453–8.586, *P* = 0.005) and antenatal proteinuria (OR = 2.127, 95% CI: 1.106–4.453, *P* = 0.045) were associated with a significantly increased risk compared with negative proteinuria. Complete regression results are presented in Table 4.

**Table 4:** Multivariate logistic regression analysis of influencing factors of hypertension during labor

Variable	B	SE	Waldχ^2^	VIF	*P*	OR(95%CI)
LDH (per 10 U/L )	0.480	0.112	19.765	1.054	< 0.001^*^	1.600 (1.290, 1.985)
Admission weight (per 5 kg)	0.430	0.105	16.169	1.040	< 0.001^*^	1.537 (1.251, 1.888)
GDM	1.450	0.472	9.446	1.012	0.002^*^	4.265 (1.691, 10.755)
Gestational proteinuria	1.262	0.453	7.751	1.046	0.005^*^	3.532 (1.453, 8.586)
Antenatal proteinuria	0.755	0.377	4.006	1.029	0.045^*^	2.127 (1.106, 4.453)

Results of the multivariate logistic regression model are presented. For continuous predictors (LDH per 10 U/L increment, admission weight per 5 kg increment), OR represents the change in risk associated with each specified unit increase. For categorical predictors (GDM, proteinuria, primiparity), the OR represents the risk compared to the reference category (absence of the condition or multiparity). All ORs are presented with their 95%CI. The VIF was examined to assess multicollinearity. *P* < 0.05 was considered statistically significant. *Abbreviations*: *LDH* lactate dehydrogenase, *GDM* gestational diabetes mellitus

The multivariate logistic regression model demonstrated favorable predictive performance, with an overall accuracy of 80.0%. Sensitivity was 77.0% (77/100) and specificity was 83.0% (83/100), indicating balanced discriminative ability. The positive predictive value was 81.9% (77/94) and the negative predictive value was 78.3% (83/106), supporting the model’s clinical utility for risk stratification. These performance metrics are detailed in Table 5.

**Table 5:** Discriminatory performance of the intrapartum hypertension prediction model

Classification Table				
Observed		Predicted		
		Labor-onset Hypertension		Percentage Correct
		No	Yes	
Labor-onset Hypertension	No	83	17	83.0
	Yes	23	77	77.0
Overall Percentage				80.0

The classification table demonstrates the model’s performance on the original dataset. Sensitivity  77/100 = 77.0%, Specificity  83/100 = 83.0%, Positive Predictive Value (PPV)  77/94 ≈ 81.9%, Negative Predictive Value (NPV)  83/106 ≈ 78.3%, Overall Accuracy  160/200 = 80.0%

### ROC curve analysis

The combined predictive model, which integrated five indicators, demonstrated excellent discriminative ability for intrapartum hypertension, with an AUC of 0.832 (95% CI: 0.757–0.873, *P* < 0.001) (Table 6).

**Table 6:** ROC Curve Analysis of combined predictive model

Area under the ROC curve				
Test Result Variable: Predicted Probability				
Area	Standard error^a^	Asymptotic Sig.^b^	Asymptotic 95% Confidence Interval	
			Lower bound	Upper bound
0.832	0.026	< 0.001	0.774	0.889

The AUC for the final multivariate model integrating all selected predictors. The asymptotic significance (*P* < 0.001) indicates that the model’s discriminative ability is significantly better than chance (AUC = 0.5)

^a^Standard error: Sampling error of the AUC estimate, measuring the precision of the statistic; smaller values indicate greater stability

^b^Asymptotic Sig.: Asymptotic P-value, testing if AUC differs from 0.5 (no predictive power). *P* < 0.001 denotes significantly better discriminative ability than chance

Among individual predictors, LDH level showed the highest predictive value (AUC = 0.731, 95% CI: 0.658–0.803, *P* < 0.001). Admission weight also demonstrated significant predictive capability (AUC = 0.707, 95% CI: 0.636–0.778, *P* < 0.001). Gestational urinary protein (AUC = 0.615, 95% CI: 0.537–0.693, *P* = 0.005) displayed modest but statistically significant predictive value. In contrast, GDM (AUC = 0.580, 95% CI: 0.501–0.659, *P* = 0.051) and antenatal urinary protein (AUC = 0.570, 95% CI: 0.491–0.649, *P* = 0.087) did not demonstrate significant discriminative ability (Table 7, Figure S1B).

**Table 7:** ROC Curve Analysis of individual predictive model

Area under the ROC curve					
Test variable	Area	Standard error^a^	Asymptotic Sig.^b^	Asymptotic 95% Confidence Interval	
				Lower bound	Upper bound
LDH	0.731	0.037	< 0.001	0.658	0.803
GDM	0.580	0.040	0.051	0.501	0.659
Gestational urinary protein	0.615	0.040	0.005	0.537	0.693
Antenatal urinary protein	0.570	0.041	0.087	0.491	0.649
Admission weight	0.707	0.036	< 0.001	0.636	0.778

The AUC for each predictor variable entered separately into a univariate logistic regression model. The asymptotic significance (*P*) indicates whether the individual predictor’s discriminative ability is significantly better than chance

^a^Standard error: Sampling error of the AUC estimate, measuring the precision of the statistic; smaller values indicate greater stability

^b^Asymptotic Sig.: Asymptotic P-value, testing if AUC differs from 0.5 (no predictive power). *P* < 0.001 denotes significantly better discriminative ability than chance

These findings indicate that while several individual factors showed significant predictive value, the combined model integrating these variables achieved superior performance in predicting intrapartum hypertension.

### Internal validation of the prediction model

The apparent AUC of the original model was 0.832 (95% CI: 0.774–0.889). Bootstrap internal validation based on 500 resamples revealed a mean optimism of 0.017 (95% CI: −0.042 to 0.075), indicating minimal overfitting (see Table 8). After optimism correction, the adjusted AUC was 0.815 (95% CI: 0.757–0.873), which remains in the good-to-excellent range for clinical prediction models. This optimism-corrected performance confirms the model’s robust discriminative ability and stability upon internal validation.

**Table 8:** Detailed bootstrap internal validation process and performance metrics for the intrapartum hypertension prediction model

Category	Item	Specification / Result
Study population	Total Sample Size	200
	Cases (Intrapartum Hypertension)	100
	Controls (Normotensive)	100
Predictor selection	Method	Lasso Regression
	Initial Candidate Variables	6 (Admission weight, Gestational Diabetes, Mid-trimester urinary protein, Antenatal urinary protein, LDH, Primiparity)
	Variables Selected for Final Model	5 (Primiparity was excluded by Lasso)
Bootstrap validation	Number of Resamples	500
	Key Steps	(1) Bootstrap sample generation; (2) Model refitting and AUC calculation within each sample; (3) Application of the refitted model to the original dataset; (4) Calculation of optimism (Bootstrap performance – Test performance); (5) Derivation of optimism-corrected AUC and 95% CI via percentile method.
Performance metrics	Apparent AUC	0.832
	Mean Optimism	0.017 (95% CI: − 0.042, 0.075)
	Optimism-Corrected AUC	0.815 (95% CI: 0.757, 0.873)
	Optimism Standard Error	0.035
Model stability assessment	Optimism Interpretation	Low optimism (0.017) indicates minimal overfitting. The 95% CI for optimism includes 0, suggesting stable performance across resamples.
	EPV Ratio	20.0, which is well above the common threshold of 10, supporting model stability.
Implications	Clinical Utility	The optimism-corrected AUC of 0.815 falls within the good range (0.8–0.9) for clinical prediction models, supporting its potential for clinical decision support.

The table summarizes the bootstrap validation procedure (500 resamples) used to correct for optimism in the model’s apparent performance. Key metrics include the apparent AUC, mean optimism, optimism-corrected AUC with its 95% confidence interval, and the Events Per Variable (EPV) ratio. Low optimism and an EPV > 10 support model stability

## Discussion

The present study developed a multivariate model for predicting intrapartum hypertension and internally validated it using bootstrap resampling. The optimism-corrected AUC of 0.815 indicates that the model retains good discriminative ability after accounting for overfitting, supporting its potential reliability for clinical application. By identifying at-risk individuals through routine mid-trimester parameters, this model reframes intrapartum hypertension not as an isolated event, but as a potential acute exacerbation within the broader spectrum of HDP. This perspective aligns with the understanding that HDP, including its intrapartum manifestations, often stems from shared underlying pathophysiological pathways such as placental dysfunction, systemic endothelial activation, and metabolic dysregulation [1, 17]. These validation steps address concerns regarding model optimism and enhance the credibility of the performance estimates.

This study developed and validated a multivariate predictive model for intrapartum hypertension that incorporates five readily accessible clinical parameters: elevated second-trimester LDH level, increased admission body weight, comorbid GDM, mid-trimester proteinuria, and antenatal proteinuria. The integrated model demonstrated robust discriminative capacity, achieving an AUC of 0.832 (95%CI: 0.757–0.873), which reflects high accuracy in identifying parturients at elevated risk for hypertension during labor. These findings suggest the potential for this model to contribute to early risk stratification; however, its clinical utility requires confirmation through external validation.

Although GDM was confirmed as a significant independent risk factor (OR = 4.265), its standalone predictive performance was relatively modest (AUC = 0.580). This indicates that the predictive value of GDM is enhanced not in isolation, but through synergistic interactions with other factors in the integrated model. GDM and HDP share common pathophysiological mechanisms, including insulin resistance, chronic inflammation, oxidative stress, and vascular endothelial dysfunction [18, 19]. The coexistence of GDM likely intensifies metabolic and cardiovascular stress during pregnancy. Hyperglycemia and hyperinsulinemia can further compromise endothelial function by reducing nitric oxide bioavailability and promoting the production of vasoconstrictors such as endothelin-1 [20]. These changes establish a pro-hypertensive environment that increases susceptibility to blood pressure elevation during the physiological stress of labor. Therefore, GDM serves as an important effect modifier, amplifying the risk associated with other predictors in the model.

Both GDM (OR = 4.265) and antenatal urinary protein (OR = 2.127) were identified as significant independent risk factors in the multivariate analysis. However, their individual predictive performance for intrapartum hypertension was relatively modest, with an AUC of 0.580 (*P* = 0.051) and 0.570 (*P* = 0.087), respectively. This indicates that, although they are important risk markers, their utility as standalone screening tools is limited. When integrated into the combined predictive model alongside other indicators such as LDH and admission weight, the overall discriminative ability of the model was significantly enhanced (AUC = 0.832) through synergistic interactions among the variables. This finding underscores the need for a comprehensive assessment of multiple risk factors rather than reliance on any single indicator. In clinical interpretation, greater emphasis should be placed on the model’s overall risk score than on the role of any individual component.

### Consideration of underlying hypertensive disorders

Our finding that elevated second-trimester LDH and proteinuria strongly predicted intrapartum hypertension raises a pertinent question: whether these cases represent new-onset intrapartum events or an acute exacerbation of an underlying, subclinical hypertensive disorder. Although strict retrospective verification of normotensive status prior to labor aimed to exclude women with overt disease, the possibility of early-stage or nonhypertensive phenotypes of placental dysfunction cannot be entirely ruled out. This aligns with the concept of a “predisease” state within the HDP continuum, where biochemical and clinical signs of placental stress may precede overt hypertension. From a clinical prediction perspective, however, the presence of these biomarkers identifies a cohort of women with ostensibly normal blood pressure who are at substantially heightened risk for acute hypertensive crises during the physiological stress of labor. This distinction is crucial for initiating targeted intrapartum surveillance, regardless of whether the ultimate postpartum diagnosis meets the criteria for preeclampsia. Thus, our model serves to flag high-risk individuals for enhanced monitoring at a critical clinical juncture.

The presence of proteinuria, even at trace levels detected during the second trimester, served as a significant predictor. This finding is supported by data indicating that abbreviated protein assessment methods, such as spot urinary protein-to-creatinine ratio or short-duration timed collections, correlate strongly with the 24-hour urinary protein excretion reference standard [21, 22]. As a hallmark of glomerular endothelial injury, proteinuria is a central diagnostic feature of preeclampsia [23]. Its early emergence, which may precede overt hypertension, reflects underlying renal impairment and systemic endothelial activation [24]. Second-trimester or antenatal proteinuria, even at mild/trace levels, indicates subclinical damage to the glomerular filtration barrier caused by placental-derived anti-angiogenic factors (e.g., sFlt1) and systemic oxidative stress in early pregnancy; this subclinical endothelial dysfunction impairs the normal hemodynamic adaptation of the maternal cardiovascular system to labor, making the parturient more susceptible to acute blood pressure elevation under the stress of uterine contractions and increased cardiac output [24, 25]. Therefore, integrating routine screening for non-overt proteinuria into standard prenatal care may provide valuable early warning, enabling a more proactive and risk-stratified management approach [25, 26]. The use of shorter-duration tests offers a practical, efficient, and cost-effective initial screening strategy, which may improve both patient adherence and clinical workflow.

LDH emerged as the strongest individual predictor (AUC = 0.731), a finding supported by strong biological plausibility. The human placenta exhibits high glycolytic activity and lactate production even under normoxic conditions [27, 28]. This glycolytic flux is significantly amplified under placental hypoxia or ischemia, a common feature of hypertensive disorders. Hypoxia-inducible factors stimulate the upregulation of LDH activity, catalyzing the conversion of pyruvate to lactate and contributing to maternal metabolic acidosis. Beyond its role in anaerobic metabolism, LDH is an established clinical marker of cellular injury and hemolysis. Its elevation in serum reflects widespread cellular damage, including hepatocyte necrosis, erythrocyte breakdown, and placental barrier dysfunction [29, 30]. Our results align with accumulating evidence that identifies LDH as a sensitive marker for preeclampsia severity, with levels correlating with the degree of systemic endothelial dysfunction and end-organ involvement. Furthermore, previous studies have documented significant positive correlations between circulating LDH levels and both systolic and diastolic blood pressures in pregnant women, supporting its role as an accessible and reliable biochemical indicator for assessing disease progression and severity [31, 32].

Admission body weight, which reflects a combination of pre-pregnancy obesity and excessive gestational weight gain, was also a strong predictor (AUC = 0.707). This relationship is underpinned by well-established metabolic changes associated with adiposity. Obesity represents a state of chronic low-grade inflammation and insulin resistance, characterized by elevated levels of pro-inflammatory cytokines (e.g., tumor necrosis factor-alpha and interleukin-6), free fatty acids, and leptin, along with reduced adiponectin levels [33, 34]. These alterations collectively contribute to endothelial dysfunction, increased systemic vascular resistance, and impaired vascular adaptation to pregnancy [35, 36]. A large-scale individual participant data meta-analysis by Santos et al., encompassing over 265,000 births, established a strong, dose-dependent association of both pre-pregnancy BMI and gestational weight gain with the risk of HDP, including preeclampsia and gestational hypertension [17]. Our results are consistent with this body of evidence and further emphasize the role of obesity-related metabolic dysregulation in the development of intrapartum hypertension, underscoring the importance of weight management before and during pregnancy.

The lack of significant differences in outcomes such as postpartum hemorrhage and NICU admission between the groups may be attributed to the relatively modest sample size and the overall low-risk profile of our study population, which limited the statistical power to detect differences in less frequent complications. Future studies with larger, higher-risk cohorts are needed to further clarify the association between intrapartum hypertension and these outcomes.

### Clinical implications for high-risk populations

The clinical impact of a predictive model is greatest when applied to specific high‑risk contexts. While derived from a predominantly low‑risk singleton cohort, our findings establish a framework that can be adapted and evaluated in populations with a higher inherent risk of HDP and its complications.

Twin pregnancies carry a substantially increased risk of preeclampsia and acute hypertensive complications, due largely to greater placental mass, higher cardiac output, and amplified hemodynamic demands. Although our prediction model was developed in singletons, the pathophysiological basis of its components, particularly those indicating metabolic load (e.g., admission weight) and potential placental dysfunction (e.g., LDH, proteinuria), remains applicable to twin pregnancies. However, absolute risk thresholds and predictor weights would likely need adjustment in this distinct population. Future research should aim to externally validate and possibly recalibrate this model in twin cohorts, or to develop twin‑specific tools that combine the biomarkers identified here with twin‑related factors such as chorionicity.

Pregnancies achieved through ART represent another clinically important high‑risk subgroup, with well‑established links to a higher incidence of hypertensive disorders of pregnancy. This association is multifactorial, likely involving underlying causes of subfertility, effects of hormonal stimulation, and alterations in placentation. A recent 2025 study comparing maternal outcomes in dichorionic diamniotic twin pregnancies further highlighted differing risk profiles between ART and spontaneously conceived pregnancies, underscoring the need for individualized monitoring strategies [37]. In our study, ART was not an independent predictor of intrapartum hypertension, which may reflect the limited number of ART cases and the strict inclusion criterion of normotension before labor. Nonetheless, the model’s use of early metabolic and biochemical markers offers a practical means of risk stratification within the growing ART population. In practice, even a moderately elevated risk score from this model in an ART pregnancy should prompt increased clinical vigilance, as it may indicate added risk on top of the baseline vulnerability associated with ART itself.

### Clinical integration and application prospects

The predictive model developed in this study relies on indicators that are readily accessible during routine prenatal care (e.g., second‑trimester laboratory tests, weight, and obstetric history), highlighting its favorable potential for clinical translation. In practice, this model could be embedded within existing electronic prenatal care systems to generate a risk assessment for pregnant women in the late third trimester (e.g., at 32–34 weeks of gestation). Individuals classified as high‑risk, for instance, those with a model‑predicted probability exceeding a predefined threshold, could then be placed on an intensified monitoring protocol. Such a protocol might include more frequent prenatal visits, antenatal fetal surveillance, planning for delivery at a facility equipped with advanced neonatal resuscitation capabilities, or management through a dedicated high‑risk obstetric clinic. For the specific high‑risk subgroups discussed previously (e.g., twin pregnancies, ART conceptions), this risk score could be incorporated into existing specialized care pathways to further tailor monitoring intensity. This stratified management approach supports the efficient allocation of healthcare resources.

Regarding implementation costs, the model itself does not impose additional testing expenses, as it utilizes data already collected in routine care. The principal “cost” lies in the increased clinical attention and resources directed toward the identified high‑risk cohort. However, this investment may be offset by the potential to avert severe intrapartum complications (such as eclampsia and placental abruption) and their associated higher treatment costs. The model is also applicable in primary care settings, where it could serve as an effective initial screening tool to assist primary care physicians in determining whether to refer a patient to a higher‑level center for closer surveillance. Its broader adoption, however, depends on successful external validation across diverse populations.

Looking ahead, the integration of novel biomarkers from emerging omics technologies, such as proteomics and metabolomics, or of continuous hemodynamic monitoring parameters, could further enhance predictive precision and advance the delivery of personalized obstetric care.

### Limitations

This study has several limitations. First, its retrospective, single-center design may have introduced selection bias and limits generalizability. The exclusion of a substantial proportion (44.8%) of identified intrapartum hypertension cases, primarily due to incomplete records, could have introduced bias if the excluded cases systematically differed from those included. Second, the study adopted a moderate sample size (200 participants, 100 cases and 100 controls), which not only limits the statistical power to detect differences in rare adverse perinatal outcomes (e.g., eclampsia, placental abruption) but also may reduce the external applicability and generalizability of the predictive model; the model’s coefficient estimates and risk stratification thresholds may be affected by the small sample size of the single-center cohort, and the results may not be directly extrapolated to other populations (e.g., different ethnic groups, high-risk obstetric populations with multiple comorbidities). Third, residual imbalances in some baseline characteristics (e.g., weight measures and parity) persisted and were subsequently adjusted for in the multivariate model. Although a standardized measurement protocol was applied, retrospective blood pressure data may still be subject to variability in timing and technique. Fourth, no formal a priori sample size calculation was performed; the moderate sample size and number of candidate predictors raise concerns about potential model overfitting. Therefore, the results should be interpreted as preliminary despite internal validation using bootstrapping. Fifth, the diagnosis of intrapartum hypertension relied on intermittent clinical blood pressure measurements, which can be influenced by factors such as pain, situational anxiety (the “white‑coat effect”), and timing relative to uterine contractions. Sixth, the study focused only on immediate perinatal outcomes and did not assess long‑term neonatal or maternal cardiovascular sequelae. Future research should address these limitations through multi-center, large-sample prospective cohort studies to externally validate and recalibrate the predictive model, including expanding the study population to include high-risk subgroups (e.g., twin pregnancies, ART-conceived pregnancies, women with pre-existing chronic diseases) to improve the model’s external validity and clinical applicability. In addition, large-sample studies can further explore the optimal risk stratification threshold of the model and its predictive value for rare severe complications of intrapartum hypertension. Future studies should address these limitations through external validation and prospective evaluation of the model’s clinical impact.

## Conclusions

In conclusion, this study developed and internally validated a multivariate prediction model for intrapartum hypertension. The model highlights several key, readily accessible clinical indicators that should alert clinicians to an elevated risk: elevated second‑trimester LDH levels, higher maternal admission weight, the presence of gestational diabetes and proteinuria (either in the second trimester or antenatally). Among these, LDH and admission weight emerged as the strongest individual predictors. Integrating these factors into a combined model demonstrated good discriminative ability, with an optimism‑corrected AUC of 0.815. For clinical practice, this tool can be applied in the late third trimester to identify women who may benefit from intensified blood pressure monitoring during labor and delivery, thereby facilitating timely intervention. Future prospective and external validation studies are needed to confirm its utility and impact on clinical outcomes.

## Supplementary Information

Supplementary Material 1. Supplementary Figure S1. ROC curves for predicting intrapartum hypertension.

## Data Availability

The datasets generated and analyzed during the current study are not publicly available due to patient privacy and ethical restrictions but are available from the corresponding author on reasonable request.
