# Supplementary material for: Development and internal validation of a predictive model for intrapartum hypertension: a retrospective case-control study
Source: BMC Pregnancy Childbirth. 2026 Mar 17;26:458. doi: 10.1186/s12884-026-08952-2 (PMC13107844; doi:10.1186/s12884-026-08952-2)
Supplement: Supplementary file 1 — Supplementary Material 1. Supplementary Figure S1. ROC curves for predicting intrapartum hypertension. [file 12884_2026_8952_MOESM1_ESM.docx]

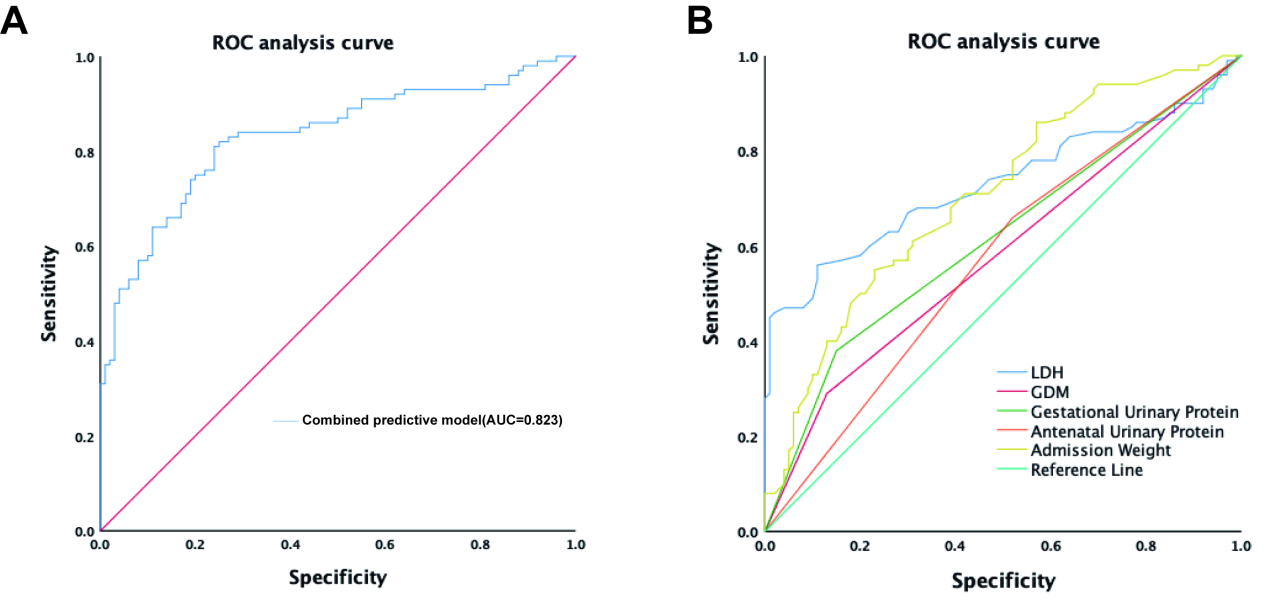


**Figure S1. ROC curves for predicting intrapartum hypertension.**

The solid blue line represents the combined multivariate prediction model (AUC = 0.863, 95% CI: 0.812–0.914). Key individual predictors are shown as dashed lines: lactate dehydrogenase (LDH, orange) , admission weight (pink). The soft pink line indicates no discriminative ability (AUC = 0.5). AUC values with 95% confidence intervals are annotated directly on the graph for immediate visual interpretation.
